# The Complex Pathway of Conventional Investigations before the Diagnosis of Functional Motor Disorders

**DOI:** 10.1002/mdc3.70340

**Published:** 2025-09-03

**Authors:** Tommaso Ercoli, Christian Geroin, Enrico Marcuzzo, Angela Sandri, Luigi Michele Romito, Roberto Eleopra, Lucia Tesolin, Francesco de Bertoldi, Alessandra Nicoletti, Giovanni Mostile, Alessandro Padovani, Andrea Pilotto, Nicola Modugno, Enrica Olivola, Benedetta Demartini, Veronica Nisticò, Roberto Erro, Sofia Cuoco, Alessandro Tessitore, Rosa De Micco, Roberto Ceravolo, Eleonora Del Prete, Carlo Dallocchio, Carla Arbasino, Francesco Bono, Cataldo Mummolo, Marcello Esposito, Assunta Trinchillo, Giovanni Fabbrini, Gina Ferrazzano, Martina Petracca, Carla Piano, Laura Bonanni, Claudia Carrarini, Alberto Albanese, Carlo Alberto Artusi, Giovanni Defazio, Paola Caruso, Giovanna Calandra‐Buonaura, Francesca Morgante, Antonio Pisani, Paolo Solla, Michele Tinazzi, Italian Registry of Functional Motor Disorders (IRFMD) study group

**Affiliations:** ^1^ Neurological Unit, AOU Sassari, University of Sassari Sassari Italy; ^2^ Department of Surgery, Dentistry, Paediatrics and Gynaecology University of Verona Verona Italy; ^3^ Neurology Unit, Movement Disorders Division, Department of Neurosciences, Biomedicine and Movement Sciences, University of Verona Verona Italy; ^4^ Fondazione IRCCS Istituto Neurologico Carlo Besta Department of Clinical Neurosciences, Parkinson and Movement Disorders Unit Milan Italy; ^5^ FND Outpatients clinic, Neurology and Stroke Unit, General Hospital of Bolzano Bolzano Italy; ^6^ Department of Psychiatry General Hospital of Bolzano Bolzano Italy; ^7^ Department G.F. Ingrassia, Section of Neurosciences University of Catania Catania Italy; ^8^ Oasi Research Institute—IRCCS Troina Italy; ^9^ Department of Clinical and Experimental Sciences, Neurology Unit University of Brescia Brescia Italy; ^10^ Laboratory of Digital Neurology and Biosensors University of Brescia Brescia Italy; ^11^ Neurology Unit, Department of Continuity of Care and Frailty, ASST Spedali Civili Brescia Hospital Brescia Italy; ^12^ Neurobiorepository and Laboratory of Advanced Biological Markers, University of Brescia and ASST Spedali Civili Hospital Brescia Italy; ^13^ Brain Health Center, University of Brescia Brescia Italy; ^14^ IRCCS Neuromed Pozzilli Italy; ^15^ Aldo Ravelli Research Center for Neurotechnology and Experimental Brain Therapeutics, Department of Health Sciences University of Milan Milan Italy; ^16^ UO Psichiatria 51 e 52, ASST Santi Paolo e Carlo, Presidio san Paolo Milan Italy; ^17^ Department of Psychology University of Milano‐Bicocca Milan Italy; ^18^ Center for Neurodegenerative Diseases (CEMAND), Department of Medicine, Surgery and Dentistry Scuola Medica Salernitana University of Salerno Baronissi Italy; ^19^ Department of Advanced Medical and Surgical Sciences University of Campania “Luigi Vanvitelli” Naples Italy; ^20^ Centro Clinico Malattie NeuroDegenerative‐Azienda Ospedaliero Universitaria Pisana University of Pisa Pisa Italy; ^21^ S.C. Neurologia, Dipartimento di Area Medica Specialistica, ASST Pavia Pavia Italy; ^22^ Center for Botulinum Toxin Therapy, Mater domini Academic Hospital Catanzaro Italy; ^23^ Clinical Neurophysiology Unit, Cardarelli Hospital Naples Italy; ^24^ S. Paolo Hospital Naples Italy; ^25^ Department Human Neurosciences Sapienza University of Rome Rome Italy; ^26^ Fondazione Policlinico Universitario Agostino Gemelli IRCCS Rome Italy; ^27^ Department of Neuroscience Università Cattolica del Sacro Cuore Rome Italy; ^28^ Department of Medicine and Aging Sciences University G. d'Annunzio of Chieti‐Pescara Chieti Italy; ^29^ IRCCS San Raffaele Rome Italy; ^30^ Department of Neurology IRCCS Humanitas Research Hospital Rozzano Italy; ^31^ Department of Neurosciences Rita Levi Montalcini University of Turin Turin Italy; ^32^ SC Neurologia 2U, AOU Città della Salute e della Scienza Torino Italy; ^33^ Department of Translational Biomedicine and Neuroscience Aldo Moro University of Bari Bari Italy; ^34^ Department of Medical Surgical and Health Sciences Cattinara Hospital University of Trieste Trieste Italy; ^35^ DIBINEM, University of Bologna Bologna Italy; ^36^ IRCCS Istituto delle Scienze Neurologiche di Bologna Bologna Italy; ^37^ City St George's University of London, Neurosciences and Cell Biology Institute London UK; ^38^ Department of Brain and Behavioral Sciences University of Pavia Pavia Italy; ^39^ IRCCS Mondino Foundation Pavia Italy

**Keywords:** functional neurological disorders, functional motor disorders, diagnosis, conventional investigations

## Abstract

**Background:**

Functional motor disorder (FMD) is a diagnosis of inclusion based on the presence of positive signs on clinical examination, and only a few tests are validated as biomarkers for FMD identification.

**Objectives:**

The aim of this study was to assess the relative frequency of different types of conventional instrumental investigations (such as magnetic resonance imaging/computed tomography [MRI/CT] scan, dopamine transporter single‐photon emission computed tomography (DaT‐SPECT), electroencephalography (EEG), neurophysiological tests, and other tests) in FMD patients before diagnosis and to identify the clinical and demographic features associated with their use.

**Methods:**

Data were obtained from the Italian Registry of Functional Motor Disorders, a multicenter initiative involving patients with a diagnosis of clinically definite FMD. Patients were consecutively enrolled at 25 Italian centers during 2 phases. Data collection initially took place between September 2018 and August 2019, and during phase 2, between January 2020 and December 2022.

**Results:**

Among the 853 patients included during the 2 phases of the registry, we identified 794 patients (93.1%) who underwent 1 or more categories of conventional investigations. Overall, conventional investigations were more likely to be performed in older FMD patients (Odds ratio [OR]: 1.02; *P* = 0.013). Interestingly, we found that more than one category of investigation was more likely to be performed in FMD patients presenting with weakness (OR: 1.85; *P* = 0.002) and with additional functional symptoms (OR: 1.54; *P* = 0.026).

**Conclusions:**

Our findings provide novel insights into the complex diagnostic process of FMD patients. This study highlights the need to identify reliable biomarkers that may help physicians diagnose FMD earlier and carefully select the most appropriate conventional investigations.

Functional motor disorders (FMD) are a common clinical manifestation of functional neurological disorders (FND),^1^ accounting for a frequent cause of stroke mimics in emergency settings and for 3%–6% of referrals to movement disorder clinics.^2^ FMD include various clinical phenotypes, such as tremor, weakness, dystonia, and gait disorders.^3^ According to the criteria proposed by Gupta and Lang, a “clinically definite” diagnosis of FMD is based on positive findings of internal inconsistency, where the motor signs may vary over time and be vulnerable to distractive maneuvers and/or incongruence, where the clinical presentation is incompatible with known neurological entities.^4^, ^5^ Further investigations may also be performed in selected cases for laboratory‐supported confirmation of the diagnosis.

Although investigations are typically normal in FMD patients and are no longer required for making the diagnosis, physicians often prescribe multiple types of conventional instrumental investigations such as magnetic resonance imaging (MRI), computed tomography (CT) scan, dopamine transporter single‐photon emission computed tomography (DaT‐SPECT), electroencephalography (EEG), neurophysiological tests, and other assessments.^6^, ^7^ This tendency to order multiple tests may originate from a concern about missing other neurological conditions, which may either coexist^8^ with or mimic FMD.^9^ Because FMD symptoms can resemble those of recognized neurological diseases, such as epilepsy, multiple sclerosis, or stroke, diagnostic uncertainty is common.^9^ Moreover, neurological comorbidities have been reported in up to 22% of patients with FMD, further complicating the diagnostic process.^8^ For instance, some studies have indicated that 2%–71% of people with a diagnosis of “epilepsy” are actually misdiagnosed, and instead, have functional seizures/spells.^10^ Likewise, misdiagnosis of multiple sclerosis is common, estimated to occur in 5%–10% of cases, with FMD frequently identified as the primary final diagnosis, followed by migraine and nonspecific white matter lesions on MRI scans.^11^ Roughly one‐quarter of stroke presentations are considered as “stroke mimics,”^12^ among which FMD, migraine, and epilepsy emerge as the most frequent final diagnoses.^12^, ^13^ There are serious implications for missing other neurological conditions; however, the consequences of failing to diagnose FMD should not be underestimated. These consequences include unnecessary treatments, delays in addressing the underlying functional disorder, and psychological harm resulting directly from the misdiagnosis, as well as high, often unnecessary healthcare costs due to excessive examinations and investigations. However, when planning investigations for FMD patients, it is essential to approach the process with clear indications, and to discuss with the patient the rationale behind this choice. Notably, the reasons for conducting conventional investigations should be communicated effectively to manage patient expectations.^7^

To date, several studies have assessed the long pathway of clinical visits made by patients before reaching the final FND diagnosis and/or the associated costs for the healthcare system.^14^, ^15^, ^16^ Many of these studies have primarily focused on FND, in particular on functional seizures/spells, reporting higher healthcare utilization due to delays in accurate diagnosis and appropriate patient management.^15^, ^17^, ^18^, ^19^ Focusing solely on FMD patients, none of the previous studies have specifically examined the types of conventional investigations in a large cohort of patients.

Therefore, the aim of the present study is to assess, in a large FMD population, the frequency of types of conventional instrumental investigations performed prior to the definite diagnosis of FMD and after the onset of FMD symptoms and to identify the clinical and demographic features associated with their use. Although this is an exploratory analysis on the type of conventional investigations, these findings may help reconsider the clinical pathway for patients with FMD, potentially leading to earlier diagnosis, and treatment of this disabling condition.

## Methods

Data were obtained from the Italian Registry of Functional Motor Disorders (IRFMD), a multicenter initiative that includes 25 Italian centers, managed by the Department of Neurosciences, Biomedicine and Movement Sciences, University of Verona, and by the Italian Academy for the Study of Parkinson's Disease and Other Movement Disorders (Accademia LIMPE DISMOV RADAC project) and Fondazione LIMPE. Approval was obtained from the Institutional Ethics Committee of the Coordinating Centre (University of Verona, Azienda Ospedaliera Universitaria Integrata Verona, Prog. 1757CESC) and confirmed by the Committees of each participating center. All patients (or their guardians) were informed about the nature of the study and gave their written consent to participate. Data collection was conducted between September 1, 2018, and August 2019, involving 410 FMD patients, previously described in other studies,^20^ and during phase 2 of the IRFMD, which took place between January 1, 2020, and December 31, 2022. The full methodology of IRFMD has been described elsewhere.^8^, ^20^, ^21^, ^22^, ^23^, ^24^, ^25^

The IRFMD involved patients with a diagnosis of clinically definite FMDs based on Gupta and Lang's criteria.^4^ Demographical and clinical variables were obtained by a structured interview that includes age, gender, FMD disease duration (time elapsing between FMD [first symptom] onset and the definite diagnosis of FMD], and neurological phenotype (tremor, weakness, dystonia, jerks, gait disorders, parkinsonism, and facial motor disorders). The registry also collected data on FMD onset (acute, defined as abrupt with deterioration within a few days or weeks; slowly progressing), patients' self‐reported nonmotor symptoms (pain, fatigue, headache, anxiety, panic attacks, insomnia, depersonalization/derealization), associated functional and somatic symptoms (functional seizures/spells, sensory symptoms, visual symptoms, cognitive disorders, fibromyalgia, irritable bowel syndrome), presence of certified psychiatric comorbidities as per a psychiatrist's diagnosis (anxiety, major depression, somatoform disorders, eating disorders, fugue state, personality disorders, post‐traumatic disorders, bipolar disorders, impulse control disorders, sexual dysfunction, gender dysphoria, and free‐entry text), certified neurological comorbidities as per a neurologist's diagnosis (migraine, Parkinson’ disease/parkinsonism, polyneuropathy, hyperkinetic movement disorders, seizures, multiple sclerosis, cerebrovascular diseases—further details are listed in Table S1), non‐neurological comorbidities as per a physician's diagnosis (heart disease, hypertension, arthritis and rheumatic diseases, tumors, thyroid disease, dyslipidemia, gastroenteric disease, diabetes mellitus), physical/psychological precipitating factors occurring before the first FMD symptom onset (psychological and physical trauma, surgery, general anesthesia, panic attack, dissociation/depersonalization, infections, and adverse drug reactions), and number of physicians seen before the diagnosis of FMD. All neurological comorbidities were diagnosed prior to the definite diagnosis of FMD.

One section of the IRFMD collected patients' self‐reported history of conventional instrumental investigations conducted after FMD symptom onset and before the definite diagnosis. The registry included predefined categories and free‐text entry: MRI, CT scan, DaT‐SPECT, EEG, neurophysiological tests (including electromyography, nerve conduction studies, and evoked potentials), and an “Other” category, which included cerebrospinal fluid analysis and other investigations commonly ordered as part of a neurologist's diagnostic work‐up, although no further specification was available in the registry. Each investigation was recorded as a binary yes/no variable, based on whether the patient reported having undergone that type of test. The number of investigations and the anatomical regions imaged were not collected. A full description of the predefined categories is provided in Table S2. All tests were ordered by different physicians not expert in FND (eg, emergency doctors, general practitioners, general neurologists). The IRFMD does not specify the setting (eg, emergency department, inpatient, outpatient) in which those investigations were conducted. Each investigation was performed after FMD symptom onset and before the definite diagnosis and was intended to clarify whether the clinical presentation was due to a functional disorder or other neurological conditions, as documented by the patient and medical record, which is consistent with standard diagnostic practice in neurological settings. For the purpose of the present study, we analyzed conventional investigations performed in the overall population of the IRFMD 1 and 2.

We first compared the patients who underwent a type of conventional investigation with those who did not. Then, we stratified and compared patients who underwent only 1 category of conventional investigation with those who underwent more than one category of investigation. Finally, to better understand the clinical pathway of patients with pure FMD, we selected from the total sample those patients without overlapping neurological disorders and with a single motor phenotype (ie, only tremor) and investigate the associated types of conventional investigation, a methodological procedure that we have already performed in another study from the IRFMD.^21^ We hypothesized that the presence of more than one FMD phenotype and neurological comorbidities might lead physicians to prescribe additional types of conventional investigations, as these factors may suggest a more heterogeneous clinical presentation.

## Statistical Analysis

Data are expressed as mean ± standard deviation for continuous variables and counts and percentages for categorical variables. For group comparisons, we used the unpaired *t* test for continuous variables and the χ^2^ or Fisher's test for categorical variables. We used a logistic regression model (the enter method) to estimate unadjusted and adjusted odds ratios (OR) and 95% confidence intervals (CIs) for the likelihood of having undergone any type of conventional instrumental investigation versus none (dependent variable), in relation to clinical and demographic characteristics (independent variables) of the IRFMD cohort. All clinically relevant variables were included simultaneously. We also tested a stepwise model, which yielded similar results (not shown), supporting the robustness of our findings. To compare the frequency distribution of conventional investigations in FMD patients with isolated phenotype and without neurological comorbidities, *Z* tests of 2 proportions or Fisher's exact tests (2 × 2) were used accordingly for the categorical ones: we applied the Bonferroni correction for multiple comparison. Statistical analyses were performed using *SPSS* statistical software (version 25; IBM‐SPSS, Armonk, NY, USA).

## Results

**Fig. 1:**
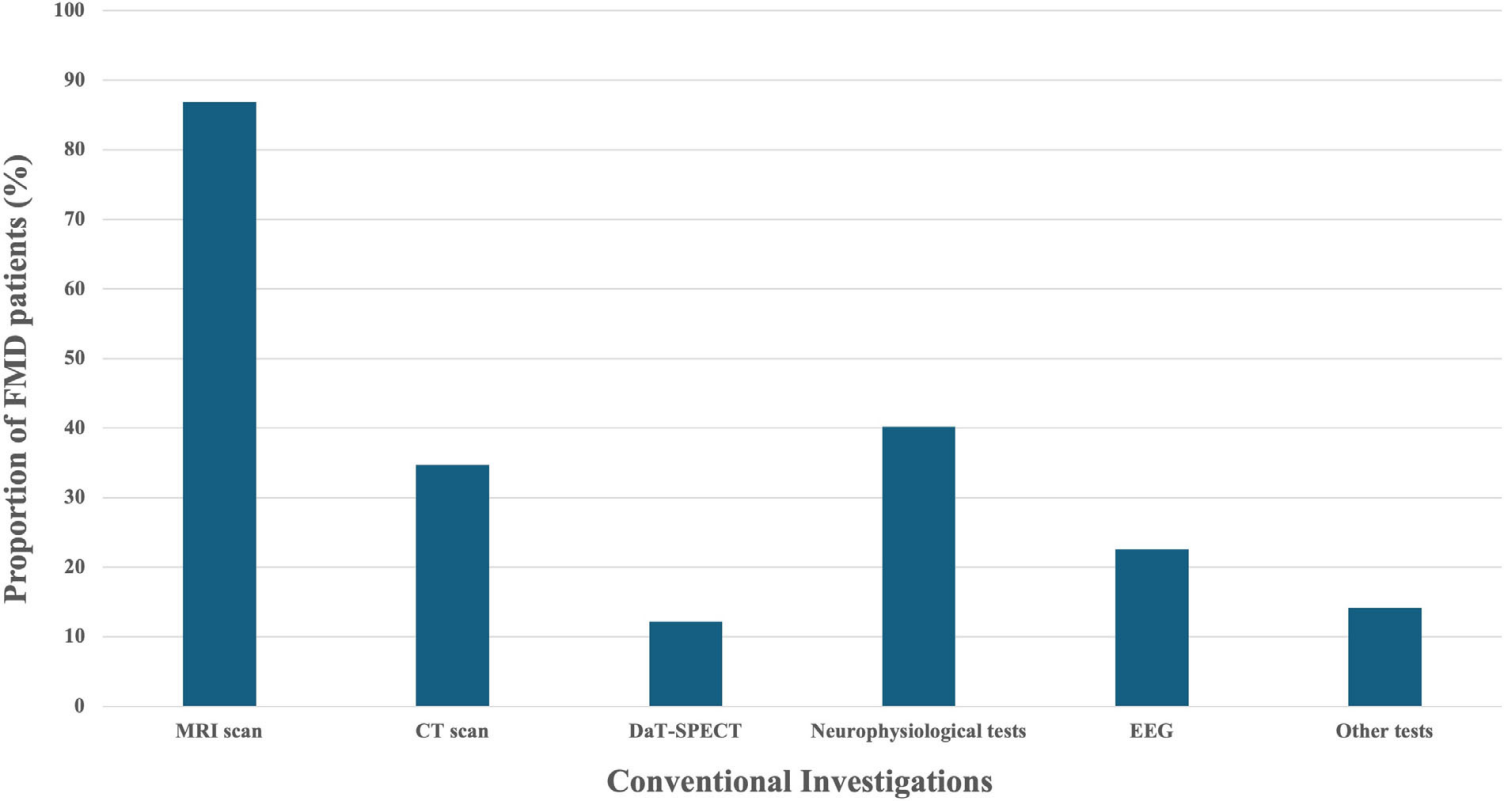
Proportion of patients who underwent a type of conventional investigations before the diagnosis of functional motor disorders (FMD) based on the type of investigation: magnetic resonance imaging (MRI) scan (ie, brain, spine, and vascular imaging such as MR angiography/MR venography [MRA/MRV]), computed tomography (CT) scan (ie, brain and spine), dopamine transporter single‐photon emission computed tomography (DaT‐SPECT), electroencephalography (EEG), neurophysiological tests (such as electromyography, nerve conduction studies, and evoked potentials), and other tests (such as cerebrospinal fluid analysis, autoimmune panels, and vitamin and nutritional deficiency screening).

Among the 853 patients included in IRFMD, we identified 794 patients (93.1%) who underwent 1 or more categories of conventional investigations before the diagnosis of FMD. The remaining 59 patients (6.9%) did not undergo any type of conventional investigation before the diagnosis of FMD. Among the 794 patients who did at least 1 category of conventional investigation, 620 (72.7%) underwent 2 or more categories of investigation. MRI emerged as the most frequently performed type of investigation. The distribution of all categories is detailed in Figure 1.

When comparing the 59 patients who did not undergo any conventional investigations with the 794 patients who did, the 2 groups were similar for many of the investigated variables (Table 1). Instead, patients who did not undergo any conventional investigations were younger than those who did (41.6 ± 18.3 years vs. 45.9 ± 16 years, *P* = 0.048), and tremor phenotype was more frequently represented in the first group (32/59 [54.2%] vs. 315/794 [39.7%], *P* = 0.028] (Table 1). Logistic regression analysis confirmed that a type of conventional investigation was more likely to be performed in older FMD patients (OR: 1.03, 95% CI: 1.01–1.05; *P* = 0.013) (Table 2).

**TABLE 1:** Comparison of demographic and clinical features of FMD patients who performed a type of conventional investigations before the diagnosis of FMD and those who did not

	FMD patients who did not undergo a type of conventional investigations (n = 59)	FMD patients who underwent a type of conventional investigations (n = 794)	*P*‐value
Female sex, n (%)	43 (72.9)	577 (72.7)	0.972
Age, yr, mean (SD)	41.6 ± 18.3	45.9 ± 16	**0.048**
FMD duration, yr, mean (SD)	4.7 ± 8.9	5.1 ± 7.03	0.755
FMD phenotype			
Tremor, n (%)	32 (54.2)	315 (39.7)	**0.028**
Weakness, n (%)	26 (44.1)	410 (51.6)	0.262
Dystonia, n (%)	10 (16.9)	209 (26.3)	0.112
Jerks, n (%)	6 (10.2)	103 (13)	0.534
Facial motor disorders, n (%)	10 (16.9)	122 (15.4)	0.746
Parkinsonism, n (%)	3 (5.1)	40 (5.0)	0.987
Gait disorders, n (%)	26 (44.1)	278 (35.0)	0.161
Acute FMD onset phenotype, n (%)	36 (63.2)	576 (73)	0.109
Self‐reported nonmotor symptoms, n (%)	52 (88.1)	694 (87.4)	0.870
Associated functional and somatic symptoms, n (%)	33 (55.9)	437 (55)	0.894
Psychiatric comorbidities, n (%)	16 (27.1)	312 (39.3)	0.64
Neurological comorbidities, n (%)	10 (16.9)	203 (25.6)	0.140
Non‐neurological comorbidities, n (%)	25 (42.4)	351 (44.2)	0.784
Precipitating factors, n (%)	29 (49.2)	424 (53.4)	0.528
MDs before the diagnosis of FMD, n, mean (SD)	2.9 ± 4.8	2.9 ± 3.5	0.942

*Note*: Bold indicates significant values; significant associations at *P* < 0.05. Abbreviations: FMD, functional motor disorders; MDs, medical doctors; SD, standard deviation.

**TABLE 2:** Clinical and demographic variables associated with the tendency to perform conventional investigations before the diagnosis of FMD

	Adjusted			
Independent variable	OR	95% CI		*P*‐value
Female vs. male sex^a^	1.23	0.64	2.36	0.538
Age, yr	1.03	1.01	1.05	**0.013**
FMD duration, yr	0.99	0.96	1.04	0.979
FMD phenotype				
Tremor, yes vs. no^a^	0.67	0.37	1.19	0.176
Weakness, yes vs. no^a^	1.79	0.96	3.34	0.067
Dystonia, yes vs. no^a^	1.94	0.93	4.03	0.077
Jerks, yes vs. no^a^	1.31	0.52	3.27	0.569
Facial motor disorders, yes vs. no^a^	0.81	0.38	1.70	0.570
Parkinsonism, yes vs. no^a^	0.92	0.26	3.27	0.901
Gait disorders, yes vs. no^a^	0.61	0.38	1.10	0.102
FMD onset				
Acute vs. chronic^a^	0.68	0.37	1.23	0.201
Self‐reported nonmotor symptoms, yes vs. no^a^	0.82	0.34	2.03	0.685
Associated functional and somatic symptoms, yes vs. no^a^	0.84	0.45	1.55	0.572
Psychiatric comorbidities, yes vs. no^a^	1.66	0.86	3.19	0.130
Neurological comorbidities, yes vs. no^a^	1.67	0.81	3.45	0.160
Non‐neurological comorbidities, yes vs. no^a^	0.93	0.50	1.73	0.827
Precipitating factors, yes vs. no^a^	1.13	0.64	1.99	0.667
MDs before the diagnosis of FMD, n	1.00	0.93	1.08	0.904

*Note*: Bold indicates significant values; significant associations at *P* < 0.05. ^a^Reference category. Abbreviations: CI, confidence interval; FMD, functional motor disorders; MDs, medical doctors; OR, Odds ratio.

When comparing the 174 FMD patients who underwent only 1 category of conventional investigation with the 620 patients who underwent more than one category, we found that the 2 groups were similar for several investigated variables (Table 3). In contrast, patients who underwent more than one category of investigation were younger than those who underwent only 1 (48.1 ± 15.7 years vs. 45.3 ± 16.1 years, *P* = 0.042); they also reported a higher frequency of weakness phenotype (62/174 [35.6%] vs. 348/620 [56.1%], *P* < 0.001] and a higher frequency of associated functional and somatic symptoms (71/174 [40.8%] vs. 366/620 [59%], *P* < 0.001). They were examined by a greater number of medical doctors before the diagnosis of FMD (2.2 ± 3 years vs. 3.1 ± 3.6 years, *P* = 0.003) (Table 3). Logistic regression analysis confirmed that undergoing more than one category of conventional investigation was more likely among FMD patients presenting with weakness (OR: 1.85, 95% CI: 1.24–2.75; *P* = 0.002) and those with associated functional and somatic symptoms (OR: 1.54, 95% CI: 1.05–2.25; *P* = 0.026) (Table 4).

**TABLE 3:** Comparison of demographic and clinical features of FMD patients who underwent one category of conventional investigation before the diagnosis of FMD and those who underwent more than one category

	FMD patients who underwent 1 category of conventional investigation (n = 174)	FMD patients who underwent more than one category of conventional investigation (n = 620)	*P*‐value
Female sex, n (%)	126 (72.4)	451 (72.7)	0.932
Age, yr, mean (SD)	48.1 ± 15.7	45.3 ± 16.1	**0.042**
FMD duration, yr, mean (SD)	5.2 ± 5.9	5 ± 7.3	0.811
FMD phenotype			
Tremor, n (%)	74 (42.5)	241 (38.9)	0.383
Weakness, n (%)	62 (35.6)	348 (56.1)	**<0.001**
Dystonia, n (%)	52 (29.9)	157 (25.3)	0.227
Jerks, n (%)	24 (13.8)	79 (12.7)	0.715
Facial motor disorders, n (%)	25 (14.4)	97 (15.6)	0.680
Parkinsonism, n (%)	5 (2.9)	35 (5.6)	0.140
Gait disorders, n (%)	54 (31)	224 (36.1)	0.213
Acute FMD onset phenotype, n (%)	118 (67.8)	458 (74.5)	0.810
Self‐reported nonmotor symptoms, n (%)	148 (85.1)	546 (88.1)	0.291
Associated functional and somatic symptoms, n (%)	71 (40.8)	366 (59)	**<0.001**
Psychiatric comorbidities, n (%)	58 (33.3)	254 (41)	0.68
Neurological comorbidities, n (%)	39 (22.4)	164 (26.5)	0.281
Non‐neurological comorbidities, n (%)	76 (43.7)	275 (44.4)	0.874
Precipitating factors, n (%)	90 (51.7)	334 (53.9)	0.616
MDs before the diagnosis of FMD, n, mean (SD)	2.2 ± 3	3.1 ± 3.6	**0.003**

*Note*: Bold indicates significant values; significant associations at *P* < 0.05. Abbreviations: FMDs, functional motor disorders; MDs, medical doctors; SD, standard deviation.

**TABLE 4:** Clinical and demographic variables associated with the tendency to perform more than one category of conventional investigation before the diagnosis of FMD

Independent variable	Adjusted			
	OR	95% CI		*P*
Female vs. male sex^a^	1.17	0.78	1.76	0.427
Age, yr	0.99	0.97	1.01	0.091
FMD duration, yr	1.01	0.97	1.03	0.851
FMD phenotype				
Tremor, yes vs. no^a^	0.96	0.66	1.41	0.847
Weakness, yes vs. no^a^	1.85	1.24	2.75	**0.002**
Dystonia, yes vs. no^a^	0.77	0.52	1.17	0.228
Jerks, yes vs. no^a^	0.93	0.55	1.57	0.783
Facial motor disorders, yes vs. no^a^	1.03	0.62	1.69	0.921
Parkinsonism, yes vs. no^a^	2.55	0.96	6.80	0.061
Gait disorders, yes vs. no^a^	1.12	0.75	1.65	0.598
FMD onset				
Acute vs. phenotype	0.74	0.50	1.11	0.145
Self‐reported nonmotor symptoms, yes vs. no^a^	0.91	0.53	1.57	0.748
Associated functional and somatic symptoms, yes vs. no^a^	1.54	1.05	2.25	**0.026**
Psychiatric comorbidities, yes vs. no^a^	1.40	0.95	2.08	0.091
Neurological comorbidities, yes vs. no^a^	1.12	0.73	1.71	0.605
Non‐neurological comorbidities, yes vs. no^a^	1.05	0.71	1.56	0.794
Precipitating factors, yes vs. no^a^	1.03	0.72	1.48	0.862
MDs before the diagnosis of FMD, n	1.07	0.99	1.16	0.055

Note: Bold indicates significant values; significant associations at *P* < 0.05. ^a^Reference category. Abbreviations: CI, confidence interval; FMDs, functional motor disorders; MDs, medical doctors; OR, odds ratio.

**Fig. 2:**
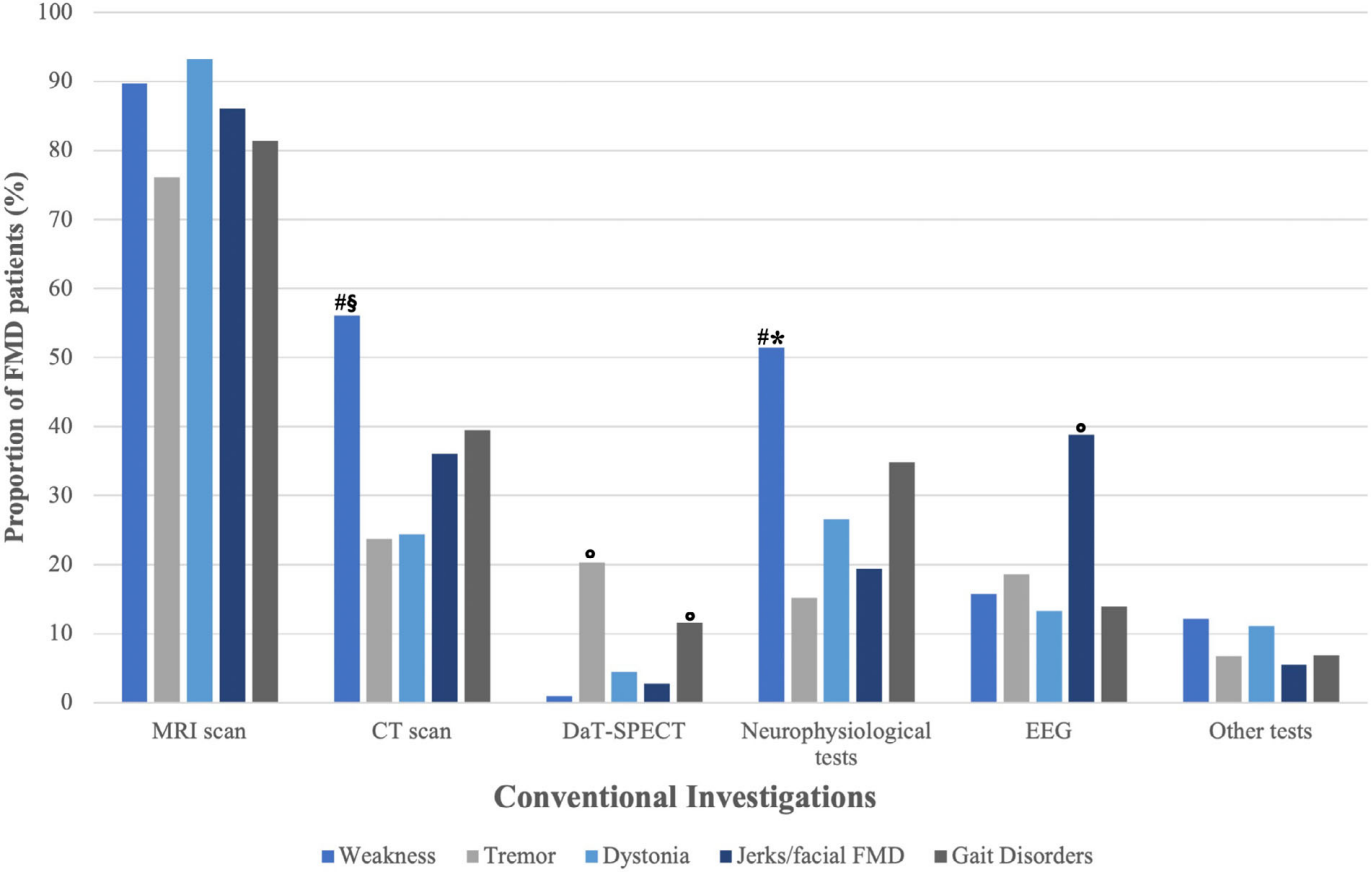
Frequency distribution of type of conventional investigations before the diagnosis of functional motor disorders (FMD) in the 290 FMD patients with isolated phenotype and without neurological comorbidities. Post hoc comparisons, *P* < 0.01: °vs. weakness; ^#^vs. tremor; ^§^vs. dystonia; *vs. jerks/facial FMD.

Focusing on FMD patients with isolated phenotype and without neurological comorbidities, we managed to select 290 patients, of whom we excluded only 2 patients with functional parkinsonism, as the number was too small to allow proper analysis. Due to their low prevalence, facial FMD patients (n = 15) were merged with jerk patients (n = 21) into a single category labeled “jerks/facial FMD.” Complete demographic and clinical features of 290 FMD patients with isolated phenotype and without neurological comorbidities are reported in Table S3. Of these 290 FMD patients, 107 (36.9%) had weakness, 59 (20.3%) suffered from tremor, 45 (15.5%) from dystonia, 43 (14.8%) from gait disorders, and 36 (12.4%) had jerks/facial FMD. Among these 5 phenotypes, patients showed similar frequencies of MRI and other tests, whereas differences emerged in the use of CT scan, DaT‐SPECT, neurophysiological tests, and EEG. Post hoc comparisons showed that CT scan was more frequently performed in patients with weakness than in patients suffering from tremor (60/107 [56.1%] vs. 14/59 [23.7%], *P* < 0.01) or dystonia (60/107 [56.1%] vs. 11/45 [24.4%], *P* < 0.01]. The proportion of patients who underwent the DaT‐SPECT was higher in the functional tremor group and in the gait disorder group compared to the ones with functional weakness (12/59 [20.3%] vs. 1/107 [0.9%], *P* < 0.01; 5/43 [11.6%] vs. 1/107 [0.9%], *P* < 0.01). Patients with jerks/facial FMD underwent EEG in greater proportion compared to patients suffering from functional weakness (14/36 [38.9%] vs. 17/107 [15.8%]). Neurophysiological tests were more frequently used in patients with weakness than those with tremor (55/107 [51.4%] vs. 9/59 [15.2%], *P* < 0.01) and jerks/facial FMD (55/107 [51.4%] vs. 7/36 [19.4%], *P* < 0.01) (Fig. 2).

## Discussion

Data from the IRFMD 1 and 2, a large multicenter initiative that collected information on Italian FMD patients, showed that only 6.9% of the patients did not undergo any type of conventional investigation before the diagnosis of FMD, such as MRI scan, CT scan, DaT‐SPECT, EEG, neurophysiological tests, and other tests. The reason why these patients did not report any type of the examinations may be attributable to several factors. For instance, FMD patients may have been directly referred to the FND clinic without first passing through the emergency department (ED), or they may have presented with clear functional semiology, leading the emergency doctor to decide not to perform further investigations in the ED. On the contrary, 93.1% of patients underwent at least 1 type of conventional investigation. Among these, 20.4% had only 1 type of conventional investigation, whereas 72.7% underwent 2 or more different types of investigations.

Although FMD should be clinically diagnosed by identifying positive signs of internal inconsistency and/or incongruence during the neurological examination, many physicians prescribe several categories of conventional investigations. The prescription of 1 or more category of conventional investigations is often driven by the aim of excluding other neurological conditions, particularly cerebrovascular disease or dopaminergic deficits, which may coexist with FMD.^8^ This picture is further complicated by recent evidence highlighting that functional symptoms may also precede the onset of other neurological conditions, especially in patients affected by Parkinson's disease.^26^

Overall, our findings indicated that older patients were more prone to undergo a type of conventional investigations, whereas no other differences were detected for the other examined variables in the whole population of the registry. Within this context, it is important to highlight that diagnosing FMD in elderly patients can be particularly challenging due to the higher prevalence of neurological comorbidities, especially cerebrovascular diseases and parkinsonism.^22^ Notably, it has also been described that hypertension was associated with older FMD onset.^22^ Moreover, compared to the general population, elderly adults face a higher risk of cerebrovascular diseases like stroke due to the presence of well‐known risk factors such as age and hypertension.^27^ In line with these observations, our results indicate that conventional investigations were more frequently performed in older FMD patients, as demonstrated by our logistic regression analysis. Additionally, certain FMD phenotypes—such as functional weakness, gait disorder, parkinsonism, jerks, and tremor—may be more frequently investigated in older adults due to the greater likelihood of these features being linked to non‐FND conditions.

Interestingly, we found that physicians tended to perform more than one category of conventional investigation in FMD patients with functional weakness, and with a greater burden of associated functional neurological symptoms. Patients with functional weakness were probably more prone to undergo several category of investigations, because this kind of symptom might be frequently misdiagnosed in emergency settings with common neurological conditions such as stroke and multiple sclerosis.^11^, ^28^ The great burden of associated functional symptoms had also an impact on the number of type of investigations, probably due to the lack of reliable biomarkers and clinical signs that allow the physician to better categorize those symptoms. Although not statistically significant, the number of physicians who had seen FMD patients before the diagnosis seemed to have a potential effect to influence the recommendation of performing investigations. Disease duration was similar in patients who underwent multiple categories of conventional investigations compared to those with only 1. We acknowledge that a longer disease duration before diagnosis could be associated with a higher number of type of tests in the absence of a diagnosis. However, as demonstrated by the multivariate analysis, the main factors influencing the prescription of investigations are not related to the time elapsed between symptom onset and FMD diagnosis but rather to factors such as the FMD phenotype—particularly weakness—and the presence of associated functional and somatic symptoms, which complicate the patient's clinical picture (Table 4).

To better describe how physicians have prescribed the type of conventional investigations before the patients reached the final diagnosis of FMD, we focused our attention on a group of patients with pure (without neurological comorbidities) and isolated FMD phenotype. We confirmed that MRI scan was the most frequently performed investigation in all the phenotypes, and it was equally distributed among groups, as well as the other types of test categories. Patients with functional weakness underwent CT scan more often than those with tremor or dystonia, probably because of its role in the differential diagnosis of suspected acute stroke. The fact that patients with functional tremor and gait disorders had a higher frequency of DaT‐SPECT than those with functional weakness should be explained as the tendency of physicians to prescribe this type of investigation when an extrapyramidal syndrome is suspected. Neurophysiological tests were more frequently reported in patients with weakness than in those with tremor or jerks, likely because these tests are routinely used to investigate suspected polyneuropathies and neuromuscular diseases.^9^ Additionally, the validated battery of tests for “laboratory‐supported” criteria in functional tremor is not yet widely used in clinical practice.^29^, ^30^

The present study describes the frequency of different categories of conventional investigations performed before the final FMD diagnosis and highlights certain patient characteristics that may lead physicians to request multiple types of tests more frequently. The high proportion of patients who performed different types of conventional investigations before the diagnosis of FMD may be due to several factors. For instance, a poor FND education in medical school may result in physicians lacking confidence in diagnosing functional symptoms using positive clinical signs during the examination.^31^ Notably, education on FND has been reported as a marginal topic in European neurology training,^32^ as well as healthcare professionals (ie, physiotherapists),^33^ and the literature mentions only a few programs specifically dedicated to the management of FND patients.^34^, ^35^ Another relevant factor is the mistaken thought that the underlying mechanism of FND/FMD is simulation.^36^ Moreover, there may also be alternative patient‐related explanations, for instance, the reluctance or failure to accept the FND/FMD diagnosis or miscommunication between patients and physicians, which led the patients to seek multiple consultations and diagnostic tests.^14^

Several studies have investigated the overall costs of FND/FMD patients on the National Health Systems in different countries, highlighting the importance of proper and timely management of these disorders. Specifically, 12 studies focused on functional seizures/spells, 4 on FND, and 2 on FMD.^15^, ^17^, ^18^, ^19^ As reported in a recent study that assessed the economic costs of delayed diagnosis of FMD in Italy, diagnostic tests performed by FMD patients had a huge impact on the National Health System, accounting for 17% of the total direct healthcare costs for patients.^14^ In the United States, the estimated annual healthcare costs for ED visits and inpatient care for patients with FND exceed $1.2 billion, compared to those of other neurologically complex disorders that require extensive diagnostic evaluation and pharmacological management.^37^ Similarly, in the UK, a recent study reported that although direct healthcare costs for patients with FND are high, their indirect costs are considerably greater.^19^ Additionally, those with a longer illness duration tend to experience higher overall costs than those with a more recent onset.^19^ Moreover, a systematic review examined the economic burden of FND, highlighting its substantial healthcare resource utilization and associated financial costs for both patients and taxpayers, along with intangible losses. Despite significant heterogeneity among the included studies, the authors suggested that interventions, such as an early and accurate diagnosis, may help reduce these costs.^15^

These findings reinforced the urgent need for the development of tailored healthcare models that can deliver comprehensive assistance to people with FND/FMD.^38^ Within this context, the Scottish 3‐stage stepped model is one of the reference systems that might enhance the care of FMD patients and may reduce healthcare costs, including those dedicated to conventional investigations. Indeed, the first step involves prompt management of FND/FMD patients by neurologists expert in FND, which may reduce the time lag for correct diagnosis and decrease the total number of physicians involved in the patients' care. The second step entails a brief therapy, typically delivered by a physiotherapist for FMDs, which can inform the neurologist about symptom inconsistency or incongruency, especially in paroxysmal symptoms that may be triggered by physical exercise.^39^ Finally, the third step includes more complex multidisciplinary care, involving the full rehabilitation team and psychiatric/psychology treatment. These steps may directly contribute to the early diagnosis of FMD, leading to a reduction in the number and type of conventional investigations prescribed for these patients.^40^

This study has limitations. Information related to conventional investigations, and the presence of neurological and psychiatric comorbidities, is based on clinical records and patient self‐reports, which may produce a recall bias. A selection bias was unlikely because patients were consecutively recruited through multicenter assessment in the entire Italian national territory and, moreover, the present study cohort shared similar clinical/demographic features to those already reported in the general FMD population. Another critical point is the fact that the precise number of time that a patient performed the same category of conventional investigation (particularly regarding MRI use) was not retrievable from the information available in the registry. Moreover, only the imaging modality was recorded, without specifying the body region examined (eg, brain vs. spine). These data would suggest further clinical predictors looking for a higher number of conventional investigations specific by type and a detailed cost analysis. Moreover, the IRFMD did not specify the setting (eg, ED, inpatient, outpatient) in which those investigations were conducted. Another limitation of this study is the absence of a control group, which restricts our ability to determine whether the frequency of conventional investigations in FMD patients differs significantly from those with other neurological conditions. Finally, we acknowledge that the category of neurological comorbidities in the IRFMD includes a heterogeneous group of conditions, from common ones like migraine and polyneuropathy to more clinically significant diseases such as multiple sclerosis, stroke, or Parkinson's disease, which likely differ in their impact on the diagnostic process. Further studies should also explore FMD patients with combined phenotypes to expand the current understanding of this important topic.

Despite the foregoing limitations, our findings provide novel insights into the diagnostic process of FMD patients, which is complex and involves the extensive use of several categories of conventional investigations. The consequences of this process may result in a delay in the diagnosis of FMD and an increase in healthcare costs. In conclusion, these findings highlight the need to determine reliable biomarkers or clinical clues that may help physicians diagnose FMD early. Further studies in different countries, which are expected to have different payers (ie, insurance) and patient populations, are needed to better characterize the use of conventional investigations in FMD patients. Moreover, future study projects should aim to collect more detailed information on the type, location, and clinical indication of each investigation to better understand the diagnostic process in patients with FMD.

## Author Roles

(1) Research project: A. Conception, B. Organization, C. Execution. (2) Statistical analysis: A. Design, B. Execution, C. Review and critique (3) Manuscript preparation: A. Writing of the first draft, B. Review and critique.

T.E.: 1A, 1B, 1C, 2A, 2B, 3A, 3B

C.G.: 1A, 1B, 1C, 2A, 2B, 3A, 3B

E.M.: 1A, 1B, 3A, 3B

A.S.: 1A, 1B, 3A, 3B

L.M.R.: 1A, 1B, 3A, 3B

R.E.: 1A, 1B, 3A, 3B

L.T.: 1A, 1B, 3A, 3B

F.D.B.: 1A, 1B, 3A, 3B

A.N.: 1A, 1B, 3A, 3B

G.M.: 1A, 1B, 3A, 3B

A.P.: 1A, 1B, 3A, 3B

A.Pi.: 1A, 1B, 3A, 3B

N.M.: 1A, 1B, 3A, 3B

E.O.: 1A, 1B, 3A, 3B

B.D.: 1A, 1B, 3A, 3B

V.N.: 1A, 1B, 3A, 3B

R.Er.: 1A, 1B, 3A, 3B

S.C.: 1A, 1B, 3A, 3B

A.T.: 1A, 1B, 3A, 3B

R.D.M.: 1A, 1B, 3A, 3B

R.C.: 1A, 1B, 3A, 3B

E.D.P.: 1A, 1B, 3A, 3B

C.D.: 1A, 1B, 3A, 3B

C.A.: 1A, 1B, 3A, 3B

F.B.: 1A, 1B, 3A, 3B

C.M.: 1A, 1B, 3A, 3B

E.Ma.: 1A, 1B, 3A, 3B

A.Tr.: 1A, 1B, 3A, 3B

G.F.: 1A, 1B, 3A, 3B

G.Fer.: 1A, 1B, 3A, 3B

M.P.: 1A, 1B, 3A, 3B

C.P.: 1A, 1B, 3A, 3B

L.B.: 1A, 1B, 3A, 3B

C.C.: 1A, 1B, 3A, 3B

A.A.: 1A, 1B, 3A, 3B

C.A.A.: 1A, 1B, 3A, 3B

G.D.: 1A, 1B, 3A, 3B

P.C.: 1A, 1B, 3A, 3B

G.C.B.: 1A, 1B, 3A, 3B

F.M.: 1A, 1B, 3A, 3B

A.Pis.: 1A, 1B, 3A, 3B

P.S.: 1A, 1B, 3A, 3B

M.T.: 1A, 1B, 1C, 2A, 2B, 3A, 3B

## Disclosures

**Ethical Compliance Statement:** The study was conducted according to the Declaration of Helsinki and to principles of good clinical practice. Approval was obtained from the Institutional Ethics Committee of the Coordinating Centre (University of Verona, Azienda Ospedaliera Universitaria Integrata Verona, Prog. 1757CESC) and confirmed by the committees of each participating center. All patients (or their guardians) were informed about the nature of the study and gave their written consent to participate. We confirm that we have read the journal's position on issues involved in ethical publication and affirm that this work is consistent with those guidelines.

**Funding Sources and Conflicts of Interest:** No specific funding was received for this work. The authors declare that there are no conflicts of interest relevant to this work.

**Financial Disclosures for the Previous 12 Months:** The authors declare that there are no additional disclosures to report.

## Supporting information


**Table S1.** Characterization of neurological/psychiatric comorbidities and precipitating factors in functional motor disorder (FMD) patients.
**Table S2.** Categories of conventional investigations recorded in the Italian Registry of Functional Motor Disorders (IRFMD).
**Table S3.** Demographic and clinical features of functional motor disorder (FMD) patients with isolated phenotype and without neurological comorbidities.

## Data Availability

The data that support the findings of this study are available from the corresponding author upon reasonable request.

## Appendix A

Coinvestigators of the Italian Registry of Functional Motor Disorders (IRFMD) study group.

**Table:**

Name	Location	Role	Contribution
Paolo Amami	Neuropsychology Unit, Fondazione IRCCS Istituto Neurologico Carlo Besta, Milan, Italy	Site investigator	Site coordinator of data acquisition
Antonio Emanuele Elia	Fondazione IRCCS Istituto Neurologico Carlo Besta, Department of Clinical Neurosciences, Parkinson and Movement Disorders Unit, Milan, Italy	Site investigator	Site coordinator of data acquisition
Nico Golfrè Andreasi	Fondazione IRCCS Istituto Neurologico Carlo Besta, Department of Clinical Neurosciences, Parkinson and Movement Disorders Unit, Milan, Italy	Site investigator	Site coordinator of data acquisition
Silvia Barca	Fondazione IRCCS Istituto Neurologico Carlo Besta, Department of Clinical Neurosciences, Parkinson and Movement Disorders Unit, Milan, Italy Unità Operativa di Medicina Interna, Ospedale Nostra Signora della Mercede di Lanusei, Lanusei, Italy	Site investigator	Site coordinator of data acquisition
Francesco Teatini	FND outpatients clinic, Neurology and Stroke Unit, General Hospital of Bolzano, Bolzano, Italy	Site investigator	Site coordinator of data acquisition
Ilaria Franch	Neurology and Stroke Unit, General Hospital of Bolzano, Bolzano, Italy	Site investigator	Site coordinator of data acquisition
Igor Florio	Neurology and Stroke Unit, General Hospital of Bolzano, Bolzano, Italy	Site investigator	Site coordinator of data acquisition
Andreas Conca	Department of Psychiatry, General Hospital of Bolzano, Bolzano, Italy	Site investigator	Site coordinator of data acquisition
Tiziana Comunale	Department of Clinical and Experimental Sciences, Neurology Unit, University of Brescia, Italy Laboratory of Digital Neurology and Biosensors, University of Brescia, Italy Neurology Unit, Department of Continuity of Care and Frailty, ASST Spedali Civili Brescia Hospital, Italy	Site investigator	Site coordinator of data acquisition
Cinzia Zatti	Department of Clinical and Experimental Sciences, Neurology Unit, University of Brescia, Italy Laboratory of Digital Neurology and Biosensors, University of Brescia, Italy Neurology Unit, Department of Continuity of Care and Frailty, ASST Spedali Civili Brescia Hospital, Italy	Site investigator	Site coordinator of data acquisition
Klaudia eshja	Department of Clinical and Experimental Sciences, Neurology Unit, University of Brescia, Italy Laboratory of Digital Neurology and Biosensors, University of Brescia, Italy Neurology Unit, Department of Continuity of Care and Frailty, ASST Spedali Civili Brescia Hospital, Italy	Site investigator	Site coordinator of data acquisition
Chiara Tolassi	Department of Clinical and Experimental Sciences, Neurology Unit, University of Brescia, Italy Laboratory of Digital Neurology and Biosensors, University of Brescia, Italy Neurology Unit, Department of Continuity of Care and Frailty, ASST Spedali Civili Brescia Hospital, Italy Neurobiorepository and Laboratory of Advanced Biological Markers, University of Brescia and ASST Spedali Civili Hospital, Brescia, Italy	Site investigator	Site coordinator of data acquisition
Francesca Conte	Department of Psychology, University of Milano‐Bicocca, Italy	Site investigator	Site coordinator of data acquisition
Diana Goeta	Aldo Ravelli Research Center for Neurotechnology and Experimental Brain Therapeutics, Department of Health Sciences, University of Milan, Milan, Italy	Site investigator	Site coordinator of data acquisition
Giovanni Broglia	Aldo Ravelli Research Center for Neurotechnology and Experimental Brain Therapeutics, Department of Health Sciences, University of Milan, Milan, Italy	Site investigator	Site coordinator of data acquisition
Paolo Barone	Center for Neurodegenerative Diseases (CEMAND), Department of Medicine, Surgery and Dentistry Scuola Medica Salernitana, University of Salerno, Baronissi (SA), Italy	Site investigator	Site coordinator of data acquisition
Maria Teresa Pellecchia	Center for Neurodegenerative Diseases (CEMAND), Department of Medicine, Surgery and Dentistry Scuola Medica Salernitana, University of Salerno, Baronissi (SA), Italy	Site investigator	Site coordinator of data acquisition
Cristiano Sorrentino	Center for Neurodegenerative Diseases (CEMAND), Department of Medicine, Surgery and Dentistry Scuola Medica Salernitana, University of Salerno, Baronissi (SA), Italy	Site investigator	Site coordinator of data acquisition
Francesco Paolo Bonifacio	Department of Advanced Medical and Surgical Sciences, University of Campania “Luigi Vanvitelli,” Naples, Italy	Site investigator	Site coordinator of data acquisition
Simone Aramini	Department of Advanced Medical and Surgical Sciences, University of Campania “Luigi Vanvitelli,” Naples, Italy	Site investigator	Site coordinator of data acquisition
Ferdinando Ambrosio	Department of Advanced Medical and Surgical Sciences, University of Campania “Luigi Vanvitelli,” Naples, Italy	Site investigator	Site coordinator of data acquisition
Davide Paoli	Centro Clinico Malattie NeuroDegenerative‐Azienda Ospedaliero Universitaria Pisana	Site investigator	Site coordinator of data acquisition
Sonia Mazzucchi	Centro Clinico Malattie NeuroDegenerative‐Azienda Ospedaliero Universitaria Pisana	Site investigator	Site coordinator of data acquisition
Daniela Frosini	Centro Clinico Malattie NeuroDegenerative‐Azienda Ospedaliero Universitaria Pisana	Site investigator	Site coordinator of data acquisition
Giovanna Savorgnan	S.C. Neurologia, Dipartimento di Area Medica Specialistica, ASST Pavia, Italia	Site investigator	Site coordinator of data acquisition
Sara Mazza	S.C. Neurologia, Dipartimento di Area Medica Specialistica, ASST Pavia, Italia	Site investigator	Site coordinator of data acquisition
Maria Paola Barillari	Center for Botulinum Toxin Therapy, Mater domini Academic Hospital, Catanzaro, Italy	Site investigator	Site coordinator of data acquisition
Leone Vadalà	Center for Botulinum Toxin Therapy, Mater domini Academic Hospital, Catanzaro, Italy	Site investigator	Site coordinator of data acquisition
Habetswallner Francesco	Clinical Neurophysiology Unit, Cardarelli Hospital, Naples, Italy	Site investigator	Site coordinator of data acquisition
Iorillo Filippo	Clinical Neurophysiology Unit, Cardarelli Hospital, Naples, Italy	Site investigator	Site coordinator of data acquisition
Matteo Costanzo	Department Human Neurosciences, Sapienza University of Rome and Department of Neuroscience, Istituto Superiore di Sanità	Site investigator	Site coordinator of data acquisition
Flavia Aiello	Department Human Neurosciences, Sapienza University of Rome	Site investigator	Site coordinator of data acquisition
Anna Rita Bentivoglio	Fondazione Policlinico Universitario Agostino Gemelli IRCCS, Rome, Italy Department of Neuroscience, Università Cattolica del Sacro Cuore, Rome, Italy	Site investigator	Site coordinator of data acquisition
Serena Fragapane	Department of Human Sciences and Quality of Life Promotion, San Raffaele University, Rome, Italy Department of Neuroscience, Università Cattolica del Sacro Cuore, Rome, Italy	Site investigator	Site coordinator of data acquisition
Mirella Russo	Department of Neuroscience, Imaging and Clinical Sciences, University G. d'Annunzio of Chieti‐Pescara, Italy	Site investigator	Site coordinator of data acquisition
Dario Calisi	Department of Neuroscience, Imaging and Clinical Sciences, University G. d'Annunzio of Chieti‐Pescara, Italy	Site investigator	Site coordinator of data acquisition
Maurizio Zibetti	Department of Neurosciences Rita Levi Montalcini, University of Turin, Turin, Italy SC Neurologia 2 U, AOU Città della Salute e della Scienza, Torino, Italy	Site investigator	Site coordinator of data acquisition
Leonardo Lopiano	Department of Neurosciences Rita Levi Montalcini, University of Turin, Turin, Italy SC Neurologia 2 U, AOU Città della Salute e della Scienza, Torino, Italy	Site investigator	Site coordinator of data acquisition
Mauro Catalan	Department of Medical Surgical and Health Sciences Cattinara Hospital, University of Trieste, Italy	Site investigator	Site coordinator of data acquisition
Paolo Manganotti	Department of Medical Surgical and Health Sciences Cattinara Hospital, University of Trieste, Italy	Site investigator	Site coordinator of data acquisition
Luisa Sambati	IRCCS Istituto delle Scienze Neurologiche di Bologna, Bologna, Italy	Site investigator	Site coordinator of data acquisition
Chiara Sorbera	IRCCS Centro Neurolesi Bonino Pulejo, 98124 Messina, Italy	Site investigator	Site coordinator of data acquisition
Piergiorgio Grillo	Department of Brain and Behavioral Sciences, University of Pavia, Italy IRCCS Mondino Foundation, Pavia, Italy	Site investigator	Site coordinator of data acquisition
Carlo Fazio	Department of Brain and Behavioral Sciences, University of Pavia, Italy IRCCS Mondino Foundation, Pavia, Italy	Site investigator	Site coordinator of data acquisition

## References

[1] Hallett M , Aybek S , Dworetzky BA , McWhirter L , Staab JP , Stone J . Functional neurological disorder: new subtypes and shared mechanisms. Lancet Neur 2022;21(6):537–550. 10.1016/S1474-4422(21)00422-1. PMC9107510 35430029

[2] Serranová T , Di Vico I , Tinazzi M . Functional movement disorder. Neurol Clin 2023;41(4):583–603. 10.1016/j.ncl.2023.02.002. 37775192

[3] Lidstone SC , Costa‐Parke M , Robinson EJ , Ercoli T , Stone J . Functional movement disorder gender, age and phenotype study: a systematic review and individual patient meta‐analysis of 4905 cases. J Neurol Neurosurg Psychiatry 2022;93(6):609–616. 10.1136/jnnp-2021-328462. 35217516

[4] Gupta A , Lang AE . Psychogenic movement disorders. Curr Opin Neurol 2009;22(1473‐6551):430–436. 19542886 10.1097/WCO.0b013e32832dc169

[5] Stone J , Burton C , Carson A . Recognising and explaining functional neurological disorder. BMJ 2020;371:m3745. 10.1136/bmj.m3745. 33087335

[6] LaFaver K , Lang AE , Stone J , et al. Opinions and clinical practices related to diagnosing and managing functional (psychogenic) movement disorders: changes in the last decade. Eur J Neurol 2020;27(6):975–984. 10.1111/ene.14200. 32153070

[7] Gilmour GS , Lidstone SC . Moving beyond movement: diagnosing functional movement disorder. Semin Neurol 2023;43(1):106–122. 10.1055/s-0043-1763505. 36893796

[8] Tinazzi M , Geroin C , Erro R , et al. Functional motor disorders associated with other neurological diseases: beyond the boundaries of “organic” neurology. Eur J Neurol 2021;28(5):1752–1758. 10.1111/ene.14674. 33300269

[9] Stone J , Reuber M , Carson A . Functional symptoms in neurology: mimics and chameleons. Pract Neurol 2013;13(2):104–113. 23468561 10.1136/practneurol-2012-000422

[10] Xu Y , Nguyen D , Mohamed A , et al. Frequency of a false positive diagnosis of epilepsy: a systematic review of observational studies. Seizure 2016;41:167–174. 10.1016/j.seizure.2016.08.005. 27592470

[11] Walzl D , Solomon AJ , Stone J . Functional neurological disorder and multiple sclerosis: a systematic review of misdiagnosis and clinical overlap. J Neurol 2022;269(2):654–663. 10.1007/s00415-021-10436-6. 33611631 PMC8782816

[12] Gibson LM , Whiteley W . The differential diagnosis of suspected stroke: a systematic review. J R Coll Physicians Edinb 2013;43(2):114–118. 10.4997/JRCPE.2013.205. 23734351

[13] Gargalas S , Weeks R , Khan‐Bourne N , et al. Incidence and outcome of functional stroke mimics admitted to a hyperacute stroke unit. J Neurol Neurosurg Psychiatry 2017;88(1):2–6. 10.1136/jnnp-2015-311114. 26319438

[14] Tinazzi M , Gandolfi M , Landi S , Leardini C . Economic costs of delayed diagnosis of functional motor disorders: preliminary results from a cohort of patients of a specialized clinic. Front Neurol 2021;12:786126. 10.3389/fneur.2021.786126. 34956066 PMC8692714

[15] O'Mahony B , Nielsen G , Baxendale S , Edwards MJ , Yogarajah M . Economic cost of functional neurologic disorders: a systematic review. Neurology 2023;101(2):e202–e214. 10.1212/WNL.0000000000207388. 37339887 PMC10351557

[16] Stephen CD , Perez DL , Chibnik LB , Sharma N . Functional dystonia: a case‐control study and risk prediction algorithm. Ann Clin Transl Neurol 2021;8:acn3.51307. 10.1002/acn3.51307. PMC8045924 33724724

[17] LaFrance WC , Benbadis SR . Avoiding the costs of unrecognized psychological nonepileptic seizures. Neurology 2006;66(11):1620–1621. 10.1212/01.wnl.0000224953.94807.be. 16769930

[18] Anderson J , Hill J , Alford M , Oto M , Russell A , Razvi S . Healthcare resource utilization after medium‐term residential assessment for epilepsy and psychogenic nonepileptic seizures. Epilepsy Behav 2016;62:147–152. 10.1016/j.yebeh.2016.06.004. 27479776

[19] O'Mahony BW , Nelson‐Sice R , Nielsen G , et al. Cross‐sectional evaluation of health resource use in patients with functional neurological disorders referred to a tertiary neuroscience centre. BMJ Neurol Open 2024;6(1):e000606. 10.1136/bmjno-2023-000606. PMC11116875 38800070

[20] Tinazzi M , Morgante F , Marcuzzo E , et al. Clinical correlates of functional motor disorders: an Italian multicenter study. Movement Disord Clin Pract 2020;7(8):920–929. 10.1002/mdc3.13077. PMC7604660 33163563

[21] Tinazzi M , Geroin C , Marcuzzo E , et al. Functional motor phenotypes: to lump or to split? J Neurol 2021;268(12):4737–4743. 10.1007/s00415-021-10583-w. 33961091 PMC8563631

[22] Geroin C , Petracca M , Di Tella S , et al. Elderly onset of functional motor disorders: clinical correlates from the Italian registry. Movement Disord Clin Pract 2024;11(1):38–44. 10.1002/mdc3.13916. PMC10828615 38291844

[23] Tinazzi M , Pilotto A , Morgante F , et al. Functional gait disorders: demographic and clinical correlations. Parkinsonism Relat Disord 2021;91(May):32–36. 10.1016/j.parkreldis.2021.08.012. 34479056

[24] Ostuzzi G , Geroin C , Gastaldon C , et al. Characterising alexithymia in individuals with functional motor disorders: a cross‐sectional analysis of the Italian registry of functional motor disorders. J Neurol Neurosurg Psychiatry 2025:jnnp‐2024‐334788. 10.1136/jnnp-2024-334788. PMC12505115 39961654

[25] Ercoli T , Tinazzi M , Geroin C , et al. Do demographic and clinical features and comorbidities affect the risk of spread to an additional body site in functional motor disorders? J Neural Transm 2022;129(10):1271–1276. 10.1007/s00702-022-02537-x. 35972697 PMC9468120

[26] Onofrj M , Russo M , Carrarini C , et al. Functional neurological disorder and somatic symptom disorder in Parkinson's disease. J Neurol Sci 2022;433:120017. 10.1016/j.jns.2021.120017. 34629180

[27] Feigin VL , Stark BA , Johnson CO , et al. Global, regional, and national burden of stroke and its risk factors, 1990–2019: a systematic analysis for the global burden of disease study 2019. Lancet Neur 2021;20(10):795–820. 10.1016/S1474-4422(21)00252-0. PMC8443449 34487721

[28] Popkirov S , Stone J , Buchan AM . Functional Neurological Disorder: A Common and Treatable Stroke Mimic. 10.1161/STROKEAHA.120.029076 32295508

[29] Schwingenschuh P , Saifee TA , Katschnig‐Winter P , et al. Validation of “laboratory‐supported” criteria for functional (psychogenic) tremor. Mov Disord 2016;31(4):555–562. 10.1002/mds.26525. 26879346

[30] Van Der Stouwe AMM , Elting JW , Van Der Hoeven JH , et al. How typical are ‘typical’ tremor characteristics? Sensitivity and specificity of five tremor phenomena. Parkinsonism Relat Disord 2016;30:23–28. 10.1016/j.parkreldis.2016.06.008. 27346607

[31] De Liège A , Carle G , Hingray C , et al. Functional neurological disorders in the medical education: an urgent need to fill the gaps. Rev Neurol 2022;178(8):788–795. 10.1016/j.neurol.2022.03.018. 35863918

[32] Serranová T , Di Vico I , Tinazzi M , et al. Functional neurological disorder in Europe: regional differences in education and health policy. Eur J Neurol 2024;31(10):e16350. 10.1111/ene.16350. 39145716 PMC11414792

[33] Edwards MJ , Stone J , Nielsen G . Physiotherapists and patients with functional (psychogenic) motor symptoms: a survey of attitudes and interest. J Neurol Neurosurg Psychiatry 2012;83(6):655–658. 10.1136/jnnp-2011-302147. 22496582

[34] Talai A , Freedman DA , Albert DVF , Pediatric Epilepsy Research Consortium . Education research: educating child neurology residents about psychogenic nonepileptic seizures: a needs assessment. Neurol Educ 2024;3(1):e200111. 10.1212/NE9.0000000000200111. 39360154 PMC11441745

[35] Finkelstein SA , O'Neal MA , Baslet G , et al. Developing a curriculum for functional neurological disorder in neurology training: questions and answers. Neurol Clin 2023;41(4):711–728. 10.1016/j.ncl.2023.02.007. 37775200

[36] Edwards MJ , Bhatia KP . Functional (psychogenic) movement disorders: merging mind and brain. Lancet Neurol 2012;11:1474–4465. 10.1016/S1474-4422(11)70310-6. 22341033

[37] Stephen CD , Fung V , Lungu CI , Espay AJ . Assessment of emergency department and inpatient use and costs in adult and pediatric functional neurological disorders. JAMA Neurol 2021;78(1):88–101. 10.1001/jamaneurol.2020.3753. 33104173 PMC7589058

[38] LaFaver K , LaFrance WC , Price ME , Rosen PB , Rapaport M . Treatment of functional neurological disorder: current state, future directions, and a research agenda. CNS Spectr 2021;26(6):607–613. 10.1017/S1092852920002138. 33280634

[39] Geroin C , Stone J , Camozzi S , Demartini B , Gandolfi M , Tinazzi M . Triggers in functional motor disorder: a clinical feature distinct from precipitating factors. J Neurol 2022;269(7):3892–3898. 10.1007/s00415-022-11102-1. 35441888 PMC9217842

[40] Healthcare Improvement Scotland. Stepped care for functional neurological symptoms . Health Improvement Scotland ; 2012. http://www.healthcareimprovementscotland.org/our_work/long_term_conditions/neurological_health_services/neurological_symptoms_report.aspx.

