# Supplementary material for: The Complex Pathway of Conventional Investigations before the Diagnosis of Functional Motor Disorders
Source: Mov Disord Clin Pract. 2025 Sep 3;12(12):2128–42. doi: 10.1002/mdc3.70340 (PMC12715358; doi:10.1002/mdc3.70340)
Supplement: Supplementary file 1 — Table S1. Characterization of neurological/psychiatric comorbidities and precipitating factors in functional motor disorder (FMD) patients. Table S2. Categories of conventional investigations recorded in the Italian Registry of Functional Motor Disorders (IRFMD). Table S3. Demographic and clinical features of functional motor disorder (FMD) patients with isolated phenotype and without neurological comorbidities. [file MDC3-12-2128-s001.docx]

**Supplementary Table 1.** Characterization of neurological/psychiatric comorbidities and precipitating factors in FMD patients.

|  | FMD patients  (n.853) |
| --- | --- |
| Neurological comorbidities:  Multiple Sclerosis, n (%)  Parkinsonism, n (%)  Hyperkinetic movement disorders, n (%)  Polyneuropathy, n (%)  Seizure, n (%)  Cerebrovascular diseases, n (%)  Migraine, n (%) | 12 (1.4)  18 (2.1)  19 (2.2)  28 (3.3)  19 (2.2)  35 (4.1)  83 (9.7) |
|  |  |
|  |  |
|  |  |
|  |  |
|  |  |
|  |  |
|  |  |
| Psychiatric comorbidities:  Schizophrenia, n (%)  Bipolar disorders, n (%)  Major depression, n (%)  Anxiety, n (%)  Impulse control disorders, n (%)  Post-traumatic disorders, n (%)  Fugue state, n (%)  Somatoform disorders, n (%)  Eating disorders, n (%)  Sexual dysfunction, n (%) Gender dysphoria, n (%)  Personality disorders, n (%) | 10 (1.2)  17 (2)  124 (14.5)  199 (23.3)  20 (2.3)  9 (1.1)  16 (1.9)  41 (4.8)  20 (2.3)  8 (0.9)  1 (0.1)  24 (2.8) |
|  |  |
|  |  |
|  |  |
|  |  |
|  |  |
|  |  |
|  |  |
| Precipitating factors:  Physical trauma, n (%)  Psychological trauma, n (%)  Surgery, n (%)  General anesthesia, n (%)  Adverse drug reactions, n (%)  Infections, n (%)  Panic attack, n (%)  Dissociation/depersonalization, n (%) | 137 (16.1)  223 (26.1)  119 (14)  56 (6.6)  51 (6)  34 (4)  15 (1.8)  14 (1.6) |

**Supplementary Table 2.** Categories of conventional investigations recorded in the IRFMD registry.

| **Registry Category** | **Included Tests** | **Notes** |
| --- | --- | --- |
| MRI | MRI brain, MRI spine, MRA, MRV | No information on body region or number of scans collected |
| CT scan | CT brain, CT spine | No information on body region or number of scans collected |
| DaT-SPECT | Dopamine transporter SPECT | PET scans were not included in the registry |
| EEG | Electroencephalography | Listed as a distinct category from neurophysiological tests |
| Neurophysiological tests | Electromyography (EMG), nerve conduction studies (NCS), evoked potentials |  |
| Other | Cerebrospinal fluid (CSF) analysis and other tests commonly included in a neurologist’s diagnostic work-up | No further detail collected; this category was predefined in the registry |

MRI: Magnetic Resonance Imaging; MRA: Magnetic Resonance Angiography; MRV: Magnetic Resonance Venography; CT: Computed Tomography; DaT-SPECT: Dopamine Transporter Single Photon Emission Computed Tomography; EEG: Electroencephalography.

**Supplementary Table 3.** Demographic and clinical features of FMD patients with isolated phenotype and without neurological comorbidities.

|  | FMD patients with isolated phenotype and without neurological comorbidities (n.290) |
| --- | --- |
| Female sex, n (%) | 201 (69.3) |
| Age, y, mean (SD) | 44.4 + 17 |
| FMD duration, y, mean (SD) | 4.4 + 7.1 |
| FMD phenotype |  |
| Tremor, n (%) | 59 (20.3) |
| Weakness, n (%) | 107 (36.9) |
| Dystonia, n (%) | 45 (15.5) |
| Jerks/facial FMD, n (%) | 36 (12.4) |
| Gait disorders, n (%) | 43 (14.8) |
| Acute FMD onset phenotype, n (%) | 221 (76.2) |
| Self-reported non-motor symptoms, n (%) | 224 (77.2) |
| Associated functional and somatic symptoms, n (%) | 137 (47.2) |
| Psychiatric comorbidities, n (%) | 96 (33.1) |
| Non-neurological comorbidities, n (%) | 111 (38.3) |
| Precipitating factors, n (%) | 147 (50.7) |
| MDs before the diagnosis, n, mean (SD) | 1.8 + 2.0 |

Abbreviations: FMD, functional motor disorders; SD, standard deviation; FNDs, functional neurological disorders; MDs, medical doctors.
